# Supplementary material for: Antibacterial Activities of Monsonia Angustifolia and Momordica Balsamina Linn Extracts against Carbapenem-Resistant Acinetobacter Baumannii
Source: Plants (Basel). 2022 Sep 12;11(18):2374. doi: 10.3390/plants11182374 (PMC9503486; doi:10.3390/plants11182374)
Supplement: Supplementary file 1 [file plants-11-02374-s001.zip › plants-1889006-supplementary.pdf]

SUPPLEMENTARY 1 [40]

TableS1. The oligonucleotides sequences for PCR.

PRIMERS	SEQUENCES	GENES	SIZES [REFERENCES]
**OXA-51 for**<br>**OXA-51 rev**	TAATGCTTTGATCGGCCTTG<br>TGGATTGCACTTCATCTTGG	blaOXA-51	353bp [1]
**OXA-23 for**<br>**OXA-23 rev**	GATGTGTCATAGTATTCGTCG<br>TCACAACAACTAAAAGCACTG	blaOXA-23	501bp [1]

*All oligonucleotides were synthesised and purified by Inqaba Biotechnical Industries, South Africa
*Y=T or C and D = A or G or T

Table S2. Thermocycling condition used in the study.

TARGETED GENES	THERMOCYCLING CONDITION	POSITIVE CONTROL
bla$_{OXA\text{-}51}$	94 °C for 4 min	*A. baumannii* ATCC® BAA-1605™ strain
		K. pneumoniae ATCC 8303 [Klebsiella pneumoniae carbapenemase (KPC) positive control]
bla$_{OXA\text{-}23}$	35 X {94 °C for 30 sec 50 °C for 30 sec 72 °C for 1 min	
	72 °C for 90 sec	*K. pneumoniae* ATCC BAA-2146 [New Delhi metallo-β-lactamase (NDM) positive control]

*min: Minute (s)

*sec: Second (s)

Reference

40. Kock, M.M.; Bellomo, A.N.; Storm, N.; Ehlers, M.M. Prevalence of carbapenem resistance genes in Acinetobacter baumannii isolated from clinical specimens obtained from an academic hospital in South Africa. *South. Afr. J. Epidemiol. Infection* **2013**, *28*, 28–32. https://doi.org/10.1080/10158782.2013.11441516.
